# Post-war (1946-2017) population health change in the United Kingdom: A systematic review

**DOI:** 10.1371/journal.pone.0218991

**Published:** 2019-07-03

**Authors:** Dawid Gondek, David Bann, Ke Ning, Emily Grundy, George B. Ploubidis

**Affiliations:** 1 Centre for Longitudinal Studies, Department of Social Science, UCL Institute of Education, University College London, London, United Kingdom; 2 Institute for Social and Economic Research, University of Essex, Colchester, United Kingdom; UCLA Fielding School of Public Health, UNITED STATES

## Abstract

We systematically reviewed the evidence on secular trends in main chronic conditions, disability and self-assessed general health among adults in the United Kingdom, as reported in primary/secondary care databases and population-based surveys. Searches were conducted separately for: (1) trends in age-standardised or age-specific prevalence of major non-communicable diseases, disability, and self-reported general health; (2) trends in health expectancy. The databases searched were MEDLINE, EMBASE/EMBASE Classic and Web of Science (all from 1946/7). The evidence was synthesised narratively. There were 39 studies reporting trends in prevalence of health conditions and 15 studies in health expectancy. We did not find evidence for improvement in the age-standardised or age-specific prevalence of any of the studied major chronic conditions over the last few decades, apart from Alzheimer's disease and other dementias. Both increasing or stable prevalence rates with simultaneous rising life expectancy support the expansion of morbidity theory, meaning that people are expected to spend a greater number of years with chronic condition(s). The evidence on disability—expressed as prevalence or health expectancy—was mixed, but also appeared to support the expansion of morbidity among those aged 65 or over. The evidence on trends in disability for younger age is lacking. Across the studied period (1946–2017), the UK population endured more years with chronic morbidity and disability, which may place a serious strain on the health care system, the economy and the society.

## Introduction

The period following World War II saw dramatically increasing life expectancy at birth in the United Kingdom, from 66.3 years in 1946 to 82.0 in 2015.[1] This was primarily due to rising longevity among older adults, with 65 year olds in 2014 having a further life expectancy of 19.7 years compared with 13 years in 1951.[1] It has to be noted, however, that despite continuous increase in life expectancy, the current improvements are not as optimistic as they were, particularly among the older population.[2] It is unclear how population health has evolved along with increasing life expectancy, for example, whether these additional years of life have been spent in good health. This issue needs to be addressed to inform public policy, for instance, to determine if older people have the capacity to remain in employment for additional years.[3]

To date, three main theories have been proposed to explain population health change: expansion of morbidity, compression of morbidity and dynamic equilibrium (Table 1). In our review, we considered two sources of evidence, which have been rarely interpreted simultaneously in the context of the theories: trends in age-standardised or age-specific prevalence rates as well as in health expectancy and total life expectancy. The most pessimistic scenario, the expansion of morbidity theory, alludes that gains in unhealthy years are greater than those in healthy ones.[4] Hence, due to rising life expectancy, stable or rising trends in age-specific prevalence rates would lead to expansion. The most optimistic theory—compression of morbidity—states that health expectancy is rising faster than total life expectancy; meaning that age-specific prevalence of morbidity is declining over time.[4] The third theory, dynamic equilibrium, proposes that there is an overall increase in prevalence and years of morbidity, which, however, has a mildly disabling effect.[5] Dynamic equilibrium would also occur when morbidity neither expands nor compresses as both mortality and morbidity are postponed by an equal number of years (see Table 1).[5] Interpreting evidence from the perspective of these theories helps to emphasise the necessity to improve population health to compensate the consequences of increasing lifespan. Hence, only reduction in morbidity over time can be considered as a positive scenario for public health.

**Table 1:** Interpretation of findings in the context of the main population health change theories in the present study.

Study findings	Interpretation
• Decrease in age-specific/age-adjusted prevalence of morbidity • Greater increase in health expectancy than in total life expectancy	Compression of morbidity
• Increase or no change in age-specific/age-adjusted prevalence of morbidity • Greater increase in total life expectancy than in health expectancy	Expansion of morbidity
• Increase or no change in age-specific/age-adjusted prevalence of mild morbidity and decrease in age-specific/age-adjusted prevalence of severe morbidity • Equal increase in health expectancy and total life expectancy	Dynamic equilibrium

The above theories have been developed around morbidity defined as disability associated with chronic conditions.[6] Hence, health expectancies are typically estimated based on a self-rated general health or an indicator if one suffers from longstanding illness or disability. This provides a limited picture of overall trends in health. Studying parallel trends of all aspects of population health change, including disability or specific chronic conditions, would lead to more precise policy recommendations, and support the design and monitoring of interventions.[7] For instance, costs of chronic conditions to the individual and society extend beyond their disabling effects, through medical costs, sub-clinical damage to health or psychosocial and stress-related burden associated with being labelled as diseased.[6] Other health measures, such as self-reported general health, has been demonstrated to be a strong predictor of mortality and disability, even while controlling for a range of objective health measures. [8, 9] This measure may capture a multifaceted nature of health—including morbidity as well as health awareness and expectations.[8, 9] Moreover, considering simultaneous trends in various measures of morbidity is essential for understanding the needs for care resources. For instance, increasing burden of chronic diseases would imply greater need for medical care, whereas rising limitations in activities (e.g. in walking or lifting) indicates a growing demand for rehabilitation, assistive technology and social services related to personal assistance as well as for a better adapted environment.[10] Hence, we refer to morbidity as an umbrella term for chronic conditions, disability, self-perceived poor health as well as other health measures commonly used to estimate health expectancy.

Overall, there is little consensus on which—if any—of these stylised scenarios best describes recent trends and, it seems that much depends on the health conditions used to operationalise morbidity and disability. Two systematic reviews of this topic have been conducted. One in the USA, which found overall declines in mild old-age disability between 1990 and 2002, whereas conflicting findings were observed for more severe long-term disability.[11] The second review was also limited to older population and period between 1991 and 2011, but did not make any geographical restrictions. It concluded that chronic morbidity measures tended to point towards expansion of morbidity, whereas disability-related measures somewhat inconsistently produced evidence for compression of morbidity. Other European studies investigating trends in the last three decades have found evidence for all three scenarios: compression in fair or poor self-perceived health[12, 13] and disability,[14] expansion of morbidity due to chronic diseases, moderate mobility limitation[15] and mild disability.[16, 17] Available non-systematic reviews of the evidence in the UK tend to emphasise the inconclusive and scattered nature of the evidence.[18, 19] The Global Burden of Disease Study (GBD), using a vast array of data sources from both published literature and primary analyses in the UK population, produced evidence supporting expansion of morbidity theory.[20, 21] It concluded that people spent more years in poor health in 2010 compared with 1990, as the increase in the number of years in good health was smaller than the rise in life expectancy at birth.[20] This was mainly due to reductions in age-specific mortality, and largely unchanged prevalence of major health conditions (weighted by an estimate of how disabling those were).[13] Nonetheless, the GBD study included information on trends only from 1990 and provided little evidence on health outcomes other than chronic conditions, such as self-rated general health or disability.

Hence, we aimed to systematically review the evidence on trends in multiple health outcomes—expressed as prevalence rates and health expectancy—including main chronic conditions as well as disability, and self-reported general health among adults in the UK during the period of 1946–2017. This would provide a more holistic picture of population health trends in the UK and further our understanding of the inconsistencies in the evidence. We included information from both primary/secondary care databases and population-based surveys, which have different strengths and sources of bias. For instance, routinely collected data may be more sensitive to introduction of screening programmes or changes over time in health awareness, as they rely on population presented to health services. Whereas, population-based surveys may mitigate this bias by studying the same, pre-defined population. On the other hand, population-based surveys are likely to report sporadic points in time, compared to routinely collected data that have information year-by-year. Triangulation of information from these sources allowed for attaining a more reliable evidence on the population health trends.[22] Our review also includes a wider period of time (1946–2017) than the previous reviews, considering long-lasting population health changes that have taken place in the UK.

## Methods

### Search strategy and selection criteria

In order to evaluate post-war trends in morbidity among adults (16 years old or older), two types of primary outcomes were retrieved: (1) estimates of trends in age-specific or age-standardised prevalence of major chronic non-communicable conditions, disability, and self-reported general health and (2) estimates of trends in health expectancy–which were not defined based on a specific health measure due to an overall small number of studies estimating health expectancy. In addition, studies of trends in incidence were retrieved as a secondary outcome. They, however, did not provide direct evidence on the theories of population health change but may help to explain current, and predict future, trends in prevalence (see S1 File for a brief summary of the evidence on incidence). In the current study, we defined morbidity as (1) chronic morbidity (i.e. a persistent condition or otherwise long-lasting in its effects), (2) disability, (3) self-reported poor health. We also included studies using a question on limiting longstanding illness or disability, typically used in health expectancy estimates. The review focused on major contributors to chronic morbidity, hence included conditions must have accounted for at least 1 per cent of disability-adjusted years of life (DALYs) in the UK from non-communicable diseases according to the Global Burden of Diseases Study 2010 (GBD) (coronary heart disease, stroke, chronic obstructive pulmonary disease, asthma, diabetes, Alzheimer`s disease migraine, cirrhosis, musculoskeletal pain).[17] Colorectal and breast cancer as well as osteoarthritis were also included, however studies of trends in prevalence have not been found. The disability measures followed a wide definition of disability by the World Health Organisation, which covers impairments (i.e. a problem in body function or structure) and activity limitations (a difficulty in executing a task or action).[7] We also included indicators of activities of daily living (ADLs), often considered as sever disability (e.g. difficulties with bathing), and instrumental activities of daily living (IADLs), an indicator of mild disability (e.g. difficulties with shopping).[7]

Measures of self-reported general health were also included as they have been found particularly useful for population health monitoring due to being highly predictive of mortality and use of health services.[23] To meet the inclusion criteria, studies: (1) drew on population-based probability samples (having longitudinal or cross-sectional design) or primary/secondary care databases and other routinely collected data, (2) were conducted in the UK, (3) were published in English. Studies providing estimates of incidence/prevalence at a single point in time were excluded.

Separate searches for health expectancy and incidence/prevalence studies were conducted by the main author (DG) (example in Table 2). In addition, reference lists of all included studies, Google Scholar and OpenGrey Repository were screened to identify any other eligible studies. The study protocol was registered with the International Prospective Register of Systematic Reviews (PROSPERO) (registration number: CRD42017069291).

**Table 2:** The search strategy in OvidSP (including EMBASE AND MEDLINE).

Search structure for studies capturing trends in the **prevalence/incidence**					
**Concept**	*Trends*	AND	*Health indicators*	AND	*Study design*
**Examples of key words**	change in incidence OR change in prevalence OR trends in incidence		self-rated health OR SRH OR stroke		cohort* OR prospective OR retrospective OR panel
Search structure for studies capturing trends in the **health expectancy**					
**Concept**	*Trends*		AND	*Health expectancy*	
**Examples of key words**	increase* OR rise* OR gain* OR difference*			health* life expectanc* OR health expectancy OR active life expectanc*	
**Searched fields**	abstracts, key words, titles, text word, keyword heading word				
**Limits applied**	13+; English only; Article only, human only, removed duplicates				
**Truncation command used**	'root word*': captures alternative word endings				

*To note*. Searches were conducted in MEDLINE (from 1946), EMBASE (1980–2017) and EMBASE Classic (1947–1973) via OvidSP interface and Web of Science (from 1946).

An asterisk (*) was used to truncate search terms.

### Data collection

Titles and abstracts were screened independently by two reviewers (DG & KN) according to exclusion/inclusion criteria. Full texts were retrieved for all citations that were included by either reviewer. The eligibility of all full texts was also assessed by two reviewers (DG & KN). Any disagreements were resolved by discussion. The key information was extracted from all publications (available upon request) by the first author (DG). In addition, the information from 20 per cent of all the publications was extracted by the second reviewer (KN) to ensure reliability and discrepancies were resolved by discussion.

### Risk of bias assessment

Two reviewers independently assessed the risk of bias within all sources of data according to the criteria broadly based on the Newcastle-Ottawa Quality Assessment Scale (see S2 File).[24] These criteria included: (1) sample being representative of the UK population; (2) high validity and reliability of outcome assessment; (3) consistency in methodology over time. Other potential biases—not related to the data source itself—were discussed in relation to each outcome. An example could be ascertainment bias resulting from the introduction of screening programmes. The evidence was considered of high quality (free of major biases) if all three criteria were met, two met criteria indicated moderate quality and evidence with one or none met criterion was considered of low quality. Tables 3–9 indicate data sources used for each outcome as well as overall quality of body of evidence. The detailed assessment of quality of each data source can be found in S3 File.

**Table 3:** The characteristics of evidence on prevalence of each health outcome, with the emphasis on size, consistency and quality.

Health outcome and data source (n studies)†	Period	Age (range)	Conclusion & consistency of findings on trends	Overall quality of evidence † †
Coronary heart disease (n = 6): QOF (n = 1)[53] HSE/S (n = 2)[25, 53] GLS/GHS (n = 1)[53] National Morbidity Survey (n = 1)[25] BRHS (n = 1)[30] CMR (n = 1)[40] THIN (n = 1)[39] MRC CFAS (n = 1)[42]	1955-6-2013-4	All	Expansion for the entire period (mixed findings on trends: stable or increasing prevalence)	High + representative of the population; + low risk of bias related to outcome assessment, combination of both self-reports and linkage of medical records; + highly comparable methodology over time; = lack of strong evidence on other biases
Stroke (n = 4): QOF (n = 1)[53] HSE/S (n = 2)[25, 53] GLS/GHS (n = 1)[53] National Morbidity Survey (n = 1)[25] MRC CFAS (n = 1)[42] GPRD (n = 1)[43]	1970-1-2013-4	All	Expansion for the entire period (mixed findings on trends: stable or increasing prevalence)	High + representative of the population; + low risk of bias related to outcome assessment combination of both self-reports and linkage of medical records; + highly comparable methodology; = lack of strong evidence on other biases

† Some studies included more than one data source.

†† Quality criteria were representativeness of the sample of the UK population; risk of bias due to outcome assessment; comparability of the methodology over time; other biases affecting comparability of trends. Meeting 3 criteria indicates high quality of evidence, 2 moderate, 1/0 low quality of evidence (high risk of bias in comparability of trends)

'+' = no risk of bias

'-' = risk of bias

' = ' = no information on risk of bias

**Table 4:** The characteristics of evidence on prevalence of each health outcome, with the emphasis on size, consistency and quality (cont.).

Health outcome and data source (n studies)†	Period	Age (range)	Conclusion & consistency of results	Overall quality of evidence † †
Lung cancer (n = 1): THIN (n = 1)[59]	2004–2012	All	Expansion for the entire period (consistent findings on trends: increase in prevalence)	High + representative of the population; + low risk of bias related to outcome assessment, based on linkage of medical records; + highly comparable methodology; = lack of strong evidence on other biases
COPD (n = 2): QRESEARCH (n = 1)[58] GPRD (n = 1)[55]	1990–2005	All	Expansion for the entire period (consistent findings on trends: increase in prevalence)	Low + representative of the population; + low risk of bias related to outcome assessment, linkage of medical records; however spirometry data not available for checking reliability of diagnoses = no information on comparability of methodology over time; - possible ascertainment bias due to introduced incentives to create and maintain a registry of patients with COPD
Asthma (n = 3): QRESEARCH (n = 1)[57] GPRD (n = 1)[56] BHPS (n = 1) [46]	1990–2005	15+	Expansion for the entire period (consistent findings on trends: increase in prevalence)	Low + representative of the population; + low risk of bias related to outcome assessment, based on linkage of medical records; however spirometry data not available for checking reliability of diagnoses = no information on comparability of methodology over time; - publicity and awareness campaigns on asthma in the lay and medical arenas, and diagnostic bias, might play a role in the reported increase in asthma prevalence

† Some studies included more than one data source.

†† Quality criteria were representativeness of the sample of the UK population; risk of bias due to outcome assessment; comparability of the methodology over time; other biases affecting comparability of trends. Meeting 3 criteria indicates high quality of evidence, 2 moderate, 1/0 low quality of evidence (high risk of bias in comparability of trends)

'+' = no risk of bias

'-' = risk of bias

' = ' = no information on risk of bias

**Table 5:** The characteristics of evidence on prevalence of each health outcome, with the emphasis on size, consistency and quality (cont.).

Health outcome and data source (n studies)†	Period	Age (range)	Conclusion & consistency of results	Overall quality of evidence † †
Alzheimer`s disease/other dementias (n = 2): MRC CFAS (n = 1)[61] ELSA (n = 1)[60]	1989–2013	50+	Compression for the entire period (consistent findings on trends: decrease in prevalence)	High + representative of older population; + low risk of bias related to outcome assessment, based on cognitive assessment; + highly comparable methodology over time; = possible non-response bias, however, it was addressed by sensitivity analyses = lack of strong evidence on other biases
Migraine (n = 1) BHPS (n = 1)[46]	1991–1998	All	Expansion (increase in prevalence)	Low + representative of the population; - no detailed information on the outcome assessment, based on self-reports; = no information on comparability of the methodology over time = lack of strong evidence on other biases
Low back pain (n = 2): Arthritis Research Campaign (n = 1)[26] Randomly selected from lists of GPs (n = 1)[27]	1956 -1997-8	18–64	Expansion for the entire period (consistent findings: increase in prevalence of less disabling back pain, no difference in prevalence of more disabling back pain)	Low—representativeness limited to the northwest region of England; - no information on possible biases due to outcome assessment, based on self-reports; - changes to mode of data collection and definitions of the outcome; = lack of strong evidence on other biases

† Some studies included more than one data source.

†† Quality criteria were representativeness of the sample of the UK population; risk of bias due to outcome assessment; comparability of the methodology over time; other biases affecting comparability of trends. Meeting 3 criteria indicates high quality of evidence, 2 moderate, 1/0 low quality of evidence (high risk of bias in comparability of trends)

'+' = no risk of bias

'-' = risk of bias

' = ' = no information on risk of bias

**Table 6:** The characteristics of evidence on prevalence of each health outcome, with the emphasis on size, consistency and quality (cont.).

Health outcome and data source (n studies)†	Period	Age (range)	Conclusion & consistency of results	Overall quality of evidence † †
Diabetes (n = 13): MRC CFAS (n = 1)[42] DIN (n = 1)[49] DARTS (n = 1)[44] Routine hospital data (Cardiff/Vale of Glamorgan) (n = 1)[50] Hospital diabetes register (n = 1)[28] THIN (n = 2)[45, 54] GPRD (n = 1)[51] HSE/S (n = 6)[47, 48, 52, 71] BRHS (n = 1)[29] GLS (n = 1)[48]	1979–1984 2013	All	Expansion for the entire period (consistent findings on trends: increase in prevalence)	High + representative of the population; + low risk of bias related to outcome assessment, combination of both self-reports and linkage of medical records; + highly comparable methodology over time; = lowered criteria for diagnosis of diabetes by fasting plasma glucose values from ≥7.8 to 7.0 mmol/l in 2000, however, the rate of increase in incidence is similar before and after 1999; = possible ascertainment bias due to, introduction of incentives for general practitioners to better detect cases (the Quality and Outcomes Framework in 2004), however sensitivity analyses showed little impact and the increase too large to be explained solely by ascertainment bias
Cirrhosis (n = 1): GPRD (n = 1)[72]	1992–2001	25+	Expansion (increase in prevalence)	Moderate + representative of the population; + no information on the outcome assessment, based on linkage of medical records; = no information on comparability of the methodology over time = lack of strong evidence on other biases
Self-rated general health (n = 2): BHPS (n = 1)[46] General practices in Leicestershire (n = 1)[37]	1981–2008	75+	Expansion (increase in prevalence)	Low + representative of the population; = no detailed information on the outcome assessment; = no information on comparability of the methodology over time = lack of evidence on other biases

† Some studies included more than one data source.

†† Quality criteria were representativeness of the sample of the UK population; risk of bias due to outcome assessment; comparability of the methodology over time; other biases affecting comparability of trends. Meeting 3 criteria indicates high quality of evidence, 2 moderate, 1/0 low quality of evidence (high risk of bias in comparability of trends)

'+' = no risk of bias

'-' = risk of bias

' = ' = no information on risk of bias

**Table 7:** The characteristics of evidence on prevalence of each health outcome, with the emphasis on size, consistency and quality (cont.).

Health outcome and data source (n studies)†	Period	Age (range)	Conclusion & consistency of results	Overall quality of evidence † †
Disability (n = 7): MRC CFAS (n = 1)[42] ELSA (n = 1)[69] General practices in Gloucestershire (n = 1)[68] General practices in Leicestershire (n = 1)[36] GHS (n = 2)[31, 48] Family Resource Survey (n = 1)[65] HSE (n = 2)[48, 73]	1979–2012	50+	Expansion 1979–1994 (consistent findings on trends: stable prevalence) Expansion 1994–2012 (mixed findings on trends)	High + representative of the population; + low risk of bias related to outcome assessment, based on self-reports; + highly comparable methodology over time; = lack of evidence on other biases

† Some studies included more than one data source.

†† Quality criteria were representativeness of the sample of the UK population; risk of bias due to outcome assessment; comparability of the methodology over time; other biases affecting comparability of trends. Meeting 3 criteria indicates high quality of evidence, 2 moderate, 1/0 low quality of evidence (high risk of bias in comparability of trends)

'+' = no risk of bias

'-' = risk of bias

' = ' = no information on risk of bias

**Table 8:** The characteristics of evidence on health expectancy, with the emphasis on size, consistency and quality.

Health outcome and data source (n studies)†	Period	Age (range)	Conclusion & consistency of results	Overall quality of evidence † †
Limiting long-standing illness or disability (n = 10): GHS/GLS (n = 7)[32–35, 41, 62, 63] UK Census (n = 2)[64, 66] MRC CFAS (n = 1)[67]	1976–2014	All	At birth: Expansion 1976–1995; expansion for 2001–2014 (consistent) Age 65/85: Expansion for the entire period (consistent)	High + representative of the population (includes institutionalised population); + low risk of bias related to outcome assessment; + consistent methodology over time; = lack of evidence on other biases.
Self-rated general health (n = 6): HSE (n = 1)[38] MRC CFAS (n = 1)[67] GHS/GLS (n = 4)[33, 41, 62, 74]	1981–2014	0, 15, 65, 85	At birth/15: Expansion 1981–1999 (consistent); expansion for 2000–2011 (inconsistent) Age 65/85: Expansion for the entire period (consistent)	High + representative of the population (includes institutionalised population); + low risk of bias related to outcome assessment; + consistent methodology over time; = lack of evidence on other biases.
Disability (n = 2): GHS/GLS (n = 1)[32] MRC CFAS (n = 1)[67]	1985–2011	65+	1980–1994: Compression among 75-year-old or older 1991–2011: Expansion	High + representative of the population (includes institutionalised population); + low risk of bias related to outcome assessment; + consistent methodology over time; = lack of evidence on other biases.

† Some studies included more than one data source.

†† Quality criteria were representativeness of the sample of the UK population; risk of bias due to outcome assessment; comparability of the methodology over time; other biases affecting comparability of trends. Meeting 3 criteria indicates high quality of evidence, 2 moderate, 1/0 low quality of evidence (high risk of bias in comparability of trends)

'+' = no risk of bias

'-' = risk of bias

' = ' = no information on risk of bias

**Table 9:** The characteristics of evidence on health expectancy, with the emphasis on size, consistency and quality (cont.).

Health outcome and data source (n studies)†	Period	Age (range)	Conclusion & consistency of results	Overall quality of evidence † †
Measures of cognition (n = 1): MRC CFAS (n = 1)^[^^67^^]^	1991–2011	65+	Compression of morbidity for females and expansion for males	High + representative of the population (includes institutionalised population); + low risk of bias related to outcome assessment, based on cognitive assessment; + consistent methodology over time; = lack of evidence on other biases.
Summary health variable (n = 1): BHPS (n = 1)^[^^46^^]^	1991–1998	20–80	Expansion of morbidity for all ages	Low + representative of the population; = no detailed information on the outcome assessment; = no information on comparability of the methodology over time; = lack of evidence on other biases.
Other health outcomes (n = 1): HSE (n = 1)^[^^41^^]^	1991–2014	25–64	Expansion of morbidity for all ages	High + representative of the population (includes institutionalised population); + low risk of bias related to outcome assessment, based on cognitive assessment; + consistent methodology over time; = lack of evidence on other biases.

† Some studies included more than one data source.

†† Quality criteria were representativeness of the sample of the UK population; risk of bias due to outcome assessment; comparability of the methodology over time; other biases affecting comparability of trends. Meeting 3 criteria indicates high quality of evidence, 2 moderate, 1/0 low quality of evidence (high risk of bias in comparability of trends).

'+' = no risk of bias

'-' = risk of bias

' = ' = no information on risk of bias

### Synthesis of evidence

Meta-analysis of trends or cumulative reporting of year-by-year estimates were not feasible due to vast differences across studies in reporting of the estimates (e.g. prevalence rates per 100,000 person years at risk, estimates of proportion of the population who have the condition, annual % change over the studied period), as well as in definition of health outcomes, and lack of information on year-by-year estimates. Hence, this review reports on consistency in the overall trends across studies, focusing on comparability of methodology over time. The evidence is summarised narratively, and trends estimates are provided throughout the study from the data sources of highest quality. Findings are reported separately for periods 1950s-1990s and 1990s-2010s as well as for each morbidity outcome, either based on health expectancy or prevalence estimates. A majority of the studies provided age-standardised estimates for entire adult population (‘All’ in Age column of Tables 3–9) or older individuals (65-year-old or older)–hence reporting of the results is focused on those two age groups.

## Results

### Study selection

**Fig 1:**
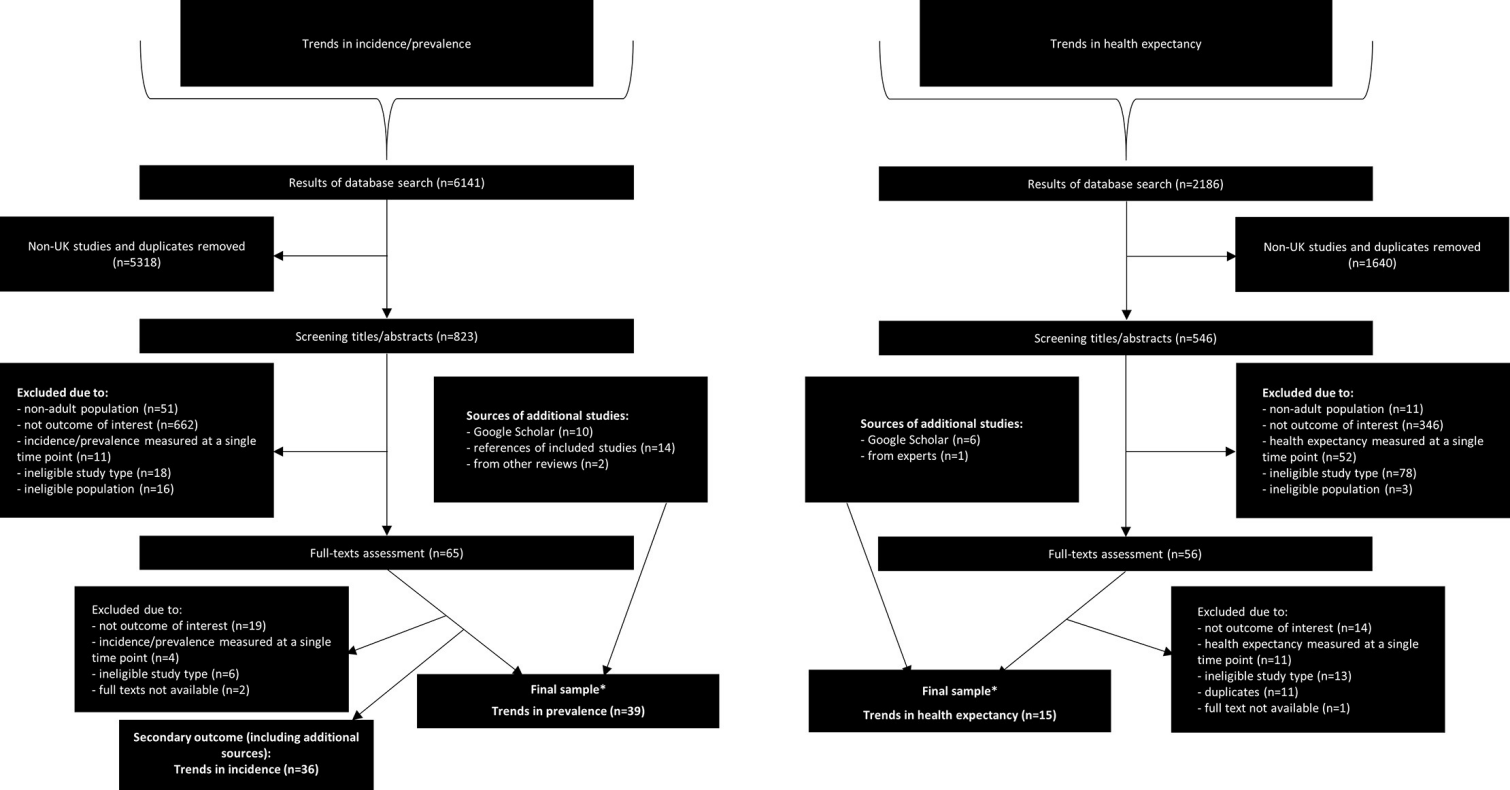
Flow chart for selection of papers and sources included in the review. *One study reported both prevalence and health expectancy.

Our literature searches identified 6141 citations examining trends in prevalence and incidence and 2186 for trends in health expectancy (see Fig 1). After removing duplicates and studies conducted outside of the UK, the initial screening of titles and abstracts was conducted by two reviewers–reaching a high agreement (Kappa = 0.78). This narrowed the number of full texts for retrieval to 65 for prevalence/incidence and 56 for health expectancy. These papers were assessed for eligibility by two reviewers (Kappa = 0.69), the main reason for exclusion was ineligibility of the outcome. Twenty papers reported incidence trends exclusively and were excluded at this stage. After inclusion of papers from other sources (see Fig 1), the total sample included 39 studies reporting trends in chronic morbidity and 15 in health expectancy.

### Period 1950s – 1990s

The trends in prevalence of chronic morbidity clearly support the expansion of morbidity scenario for the period of 1970s-1990s, across all ages and sexes. There was evidence pointing towards expansion in coronary heart diseases occurring from as early as 1950s (see Tables 3–7). Similarly, the evidence consistently points towards expansion in limiting longstanding morbidity (including illness or disability), self-reported general health at birth and among older people for the period of 1970s-1990s, and of more severe disability (with stable prevalence trends) between early 1980s and mid-1990s (see Tables 3–9). However, there is some evidence that more severe disability may have declined among the oldest participants (75 or older) between early 1980s and mid-1990s, supporting compression of morbidity for that period (see Tables 3–9).

#### Chronic morbidity

All included studies for this period reported trends in prevalence rates, rather than in health expectancy. Studied chronic conditions were coronary heart disease, stroke, musculoskeletal pain and diabetes. The National Morbidity Survey, including a representative sample of General Practitioners (GPs), reported increasing prevalence of angina from mid-1950s to 1990s, particularly among those aged 65 or older (e.g. it increased by 128% for the period of 1972–1992) and of stroke between 1971 and 1991 in all age and sex groups (increased by 40%).[25] The overall trends in the prevalence of myocardial infarction were inconsistent: prevalence rates increased slightly between 1971 and 1981 (by 15%) and subsequently fell between 1981 and 1991 (by 31%).[25] One study also reported higher point prevalence of self-reported musculoskeletal pain (e.g. cervical, dorsal) in 1994–95 compared with 1956–58.[26] However, population representativeness of the study was limited to a small north-eastern region of England and the measure of musculoskeletal pain as well as demographic characteristics somewhat varied between the surveys.[26] In another study—representative for the population and with identical methodology over time—sex- and age-standardised prevalence of self-reported back pain (lasting for at least 24 hours in previous 12 months) increased between 1987-8 and 1997-8 (by 12% in absolute terms).[27] However, severe back pain (self-reported ability to put on hosiery) fell minimally by 0.7% (-0.1%, 1.5%) among 29-59-year-olds.[27] The increase in age- and sex-standardised prevalence was also seen in diabetes between 1978 and 1996 in two large surveys conducted in primary care practices.[28, 29] According to the British Regional Heart Study, which is a representative cohort of 7722 British males aged 40–59, the annual age-adjusted rate of diabetes rose by 4.3% (0.4%, 8.2%) in the period 1978–1985 and 5.5% (3.0%, 8.1%) in 1985–1992.[29] In the same study, lifetime prevalence of coronary heart disease stayed stable in the period 1978–1985 and increased (annual % change in odds: 1.9%, 0.5% to 3.3%) in 1985–1992.[30]

#### Limiting longstanding morbidity

The proportion of older individuals (age 60–89) reporting a limiting longstanding illness, disability or infirmity in the General Household Survey increased from late-1970s until late 1980s, and tailed off in the 1990s –averaging at 42%.[31] Due to rising life expectancy, this resulted in an increase of over three years in life-time expectancy with limiting long-term ill-health.[32] Greater increase in life expectancy than the total number of years without a limiting long-standing illness or disability was also seen in other studies for overlapping period, both at birth and at age 65 or over.[33–35]

#### Disability

The analysis of the General Household Survey showed largely stable rates in ADLs (i.e. self-reported ability to independently bath, shower or wash all over) and mobility measures (i.e. self-reported ability to manage stairs and steps)—among aged 75 or older—between 1980 and 1994.[31, 32] However, there was evidence for reductions in disability among participants 85 years old or older, particularly among males.[31, 32] For instance, 18% of males reported one or more ADLs in 1994 compared with 31% in 1980.[32] The increase in health expectancy was comparable with the total life expectancy—hence overall number of years expected to be lived with disability remained stable.[32] A repeated cross-sectional survey including only 75 years old or older population of Melton Mowbray (Leicestershire, UK), also found decrease in age-adjusted prevalence of most ADLs (i.e. getting in and out of bed/a chair, dressing, getting to and from the toilet, bathing) between 1981 and 1988.[36] However, the study may be not representative for the UK population.

#### Self-rated general health

Life expectancy increased more than health expectancy with self-rated ‘good’ or ‘fairly good’ health—by 0.4 years—for those aged 65 for the period between 1981 and 1995.[33] Likewise, more recently born cohorts had a greater proportion of elderly people who rated their health as ‘less than good’ between 1981 and 1988 (e.g. by 17% among those aged 75–81).[37] The estimates were also robust to effects of migration over time.[37] Similar results were found for the period of 1981–1995 for health expectancy at birth, when the expected number of years lived in poor health increased by 1.3 years,[33] and for 1994–1999 among individuals aged 15 –with the increase by 0.9 years.[38]

### Period 1990s – 2010s

**Fig 2:**
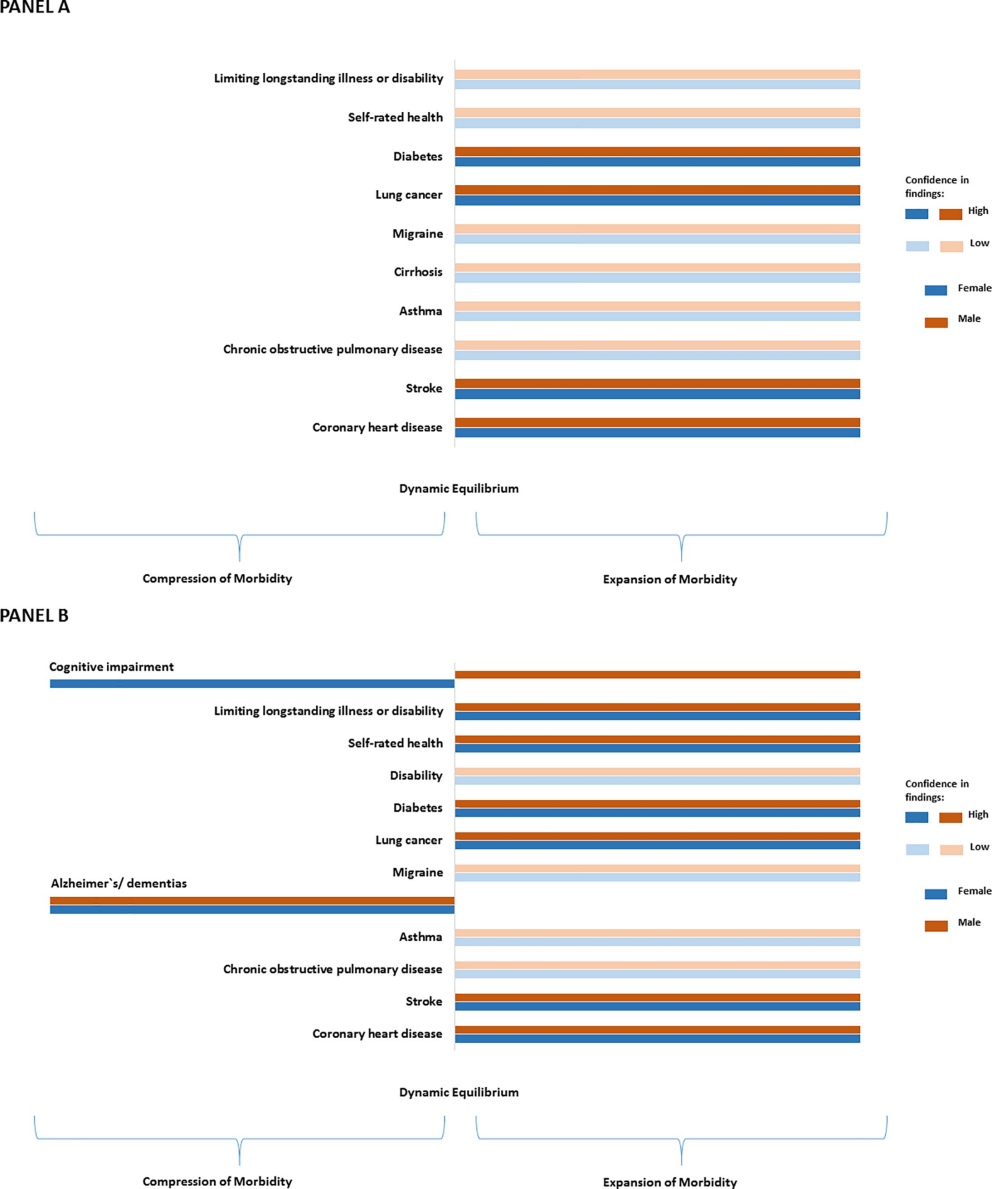
**Summary of the evidence for the period (1990–2010), for those aged 16–64 (Panel A) and 65 or older (Panel B).** Each bar indicates compression/dynamic equilibrium/expansion of morbidity in a given health condition for either males or females. Darker shades represent high confidence in findings, with evidence being consistent and of high quality (see Tables 3–9). Lighter shades represent inconsistent evidence or of low quality.

The trends in chronic morbidity consistently pointed towards expansion of morbidity across ages with rising prevalence in all studied conditions except for dementias, especially Alzheimer’s disease (decreasing trend in prevalence in 1990–2010) and coronary heart disease (stable in 2000–2010) (see Tables 3–7 and Fig 2). The expansion of morbidity was apparent due to long-standing illness or disability (as a self-reported one item indicator)—particularly among those aged 65 or older, where increase in health expectancy did not compensate for the increase in total life expectancy. Overall, expansion of morbidity in disability was also more apparent among older population (65-year-old or older), however there was a considerable inconsistency in the evidence with trends varying depending on specific measures used. Studies on disability trends among younger population were lacking. There was a clear trend for expansion of morbidity in self-reported general health for the entire period and across all ages and sexes. Finally, we found compression of morbidity for cognitive impairment among females (but expansion among males).

#### Chronic morbidity

All included studies reported trends in prevalence rates, rather than in health expectancy. Chronic conditions found for this period were coronary heart disease, stroke, lung cancer, COPD, asthma, Alzheimer`s disease and other dementias, migraine, cirrhosis, low back pain and diabetes.

According to the British Regional Heart Study, the age-standardised prevalence of coronary heart disease declined minimally (the trend was not statistically significant) among 40–59 year-old males in the period 1992–1996 (annual % change in odds: -1.4%, -3.0% to 0.2%).[30] High quality evidence—based on large primary/secondary care databases and population-based surveys, with consistent methodology over time—indicated increasing age-standardised prevalence rates between mid-1990s and mid-2000s in all ages and sexes for: coronary heart diseases,[25, 39–41] stroke[25, 41–43] and diabetes.[28, 29, 44–52] Based on large primary care databases—the Health Improvement Network (THIN) and the General Practice Research Database (GPRD)—average percentage change in age-standardized prevalence of coronary heart disease increased by 1.5% for the period 1996–2005;[39] unstandardised stroke prevalence increased by 13%, from 6.40/1000 in 1999 to 7.20/1000 in 2008;[43] and crude prevalence of diabetes prevalence increased from 2.8% in 1996 to 4.3% in 2005 (age- and sex-standardisation did not make a substantial difference).[45]

More recently, studies of trends in coronary heart disease between early-2000s and early- 2010s, based on both general practice records (the Quality and Outcomes Framework; THIN) and population-representative annual cross-sectional surveys (the National Health Surveys and the General Household Survey), concluded that the prevalence of coronary heart disease remained largely stable,[25, 30, 39, 40, 53] whereas rates of stroke[53] and diabetes[54] increased (respectively by 13% and 123% in relative terms).

Similarly, high-quality evidence, based both on primary/secondary databases and population-based surveys, indicated an increase in prevalence of chronic respiratory diseases (asthma and chronic obstructive pulmonary disease) from early 1990s to mid-2000s.[46, 55–58] For instance, according to the QRESEARCH, there was a relative increase in the standardised prevalence of COPD (by 24%) and of asthma (by 20%) between 2001 and 2005.[57, 58] The British Household Panel Survey (BHPS) also reported an increase in self-reported migraine between 1991 and 1998 in both males and females across most of the age groups, particularly 65 or older.[46] A study using THIN showed that age- and sex-standardised prevalence rates of lung cancer rose by 23% between 2004 and 2012, mainly due to increases among females.[59] We found two high-quality prospective longitudinal studies, the English Longitudinal Study of Ageing (ELSA) and the Medical Research Council Cognitive Function and Ageing Studies (MRC CFAS I and II), both representative of the older population, aged 50 or older and 65 or older respectively.[60, 61] Both studies showed a relative decrease in age and sex-standardised prevalence of dementia by 30% for the period of 2002/2003-2012/3 and by 40% for 1989–2011.[60, 61]

#### Limiting longstanding morbidity

Large population-based surveys, representative for the UK population and with consistent methodology over time (GHS/GLS, the Integrated Household Survey, Continuous Household Survey and the UK census data), all found that the total life expectancy has increased by a greater number of years than life expectancy without a limiting longstanding illness or disability at age 65 or older for the period between early-1990s and early-2010s.[62–64] For instance, estimates based on the National Census showed increase by 0.4 years for 85-year-olds between 1991 and 2001.[64] However, there is some evidence that between 2002 and 2012, those born after 1924 experienced lower odds of having long-standing illness, disability or infirmity, whereas the prevalence was stable for females.[65] However, among people who reported longstanding morbidity, the number of disabilities increased (e.g. mobility, manual dexterity) for each successive cohort (incidence rate ratio 1.03), suggesting greater severity of morbidity.[65]

The findings for trends at younger ages were inconsistent, however overall pointing towards a greater increase in total life expectancy than health expectancy. Wohland and colleagues [64] found a total life expectancy increased by 1.3 years more than health expectancy in the period of 1991–2001. Similar findings were obtained for a shorter period of 2001–2011, based on the UK National Census data from London only (expansion of morbidity by 1.7 years).[66] On the contrary, estimates based on the GHS/GLS showed a greater increase in health expectancy than total life expectancy—for males only (with no difference among females)—in the shorter period of 2000–2011 (by 0.9 year).[62, 63] Finally, a study based on repeated annual cross-sections of the Health Survey for England from 1991 to 2014 (participants aged 25–64), found an equal increase in health expectancy and total life expectancy.[41]

#### Disability

There was an increase of 2.5 years with self-reported ADLs (e.g. putting on shoes and socks) and IADLs (e.g. shopping) in the period of 1991–2011 among 65-year-old.[67] The findings were somewhat mixed among studies that provided age-standardised prevalence rates. These inconsistencies were found despite overlapping study periods, comparable populations and definitions of disability. The analysis of the Health Survey for England indicated increasing rates of severe disability (e.g. washing/bathing, dressing) among 65-year-olds, up from 13.5% in 1995 to 15.3% in 2001.[48] By contrast, the results from the General Household Survey indicated a decline in severe disability rates among people aged 65 and over, from 21 per cent in 1994–95 to 18 per cent in 2001–2.[48] The reasons for the diverging results are not clear. In studies with multiple indicators of disability, the trends were also mixed–regardless of severity of disability or type of activity. For instance, a repeated cross-sectional study in England, conducted between 1998 and 2008, found reductions in sex-standardised disability rates among those aged 75 or older in outdoor mobility, washing difficulty, ability to prepare a meal, joint pain and requiring help with nail care, whereas differences were not found in dressing difficulty, indoor mobility and prevalence of falls.[68] Similarly, a study using the HSE—conducted among 65 years and older in 1992–2007—found decreasing age-adjusted prevalence in usual activities, stable rates in limitations in self-care activities and increasing rates in limitations in walking 200 yards and climbing stairs. Chatterji and colleagues found declining proportion of people aged 50–75, but not 75 or older, with severe disability (ADLs) in 2002–2008.[69] Whereas, proportion of individuals with mild disability (IADLs) increased over the period across all ages, with a sharper rise among 75-year-old or older.[69] In the only study with younger population, a greater proportion of 25-year-old or older reported problems or disability related to arms, legs, hands, feet, back or neck.[46]

#### Self-rated general health

The findings somewhat consistently showed that life expectancy increased more than expected years with self-reported ‘good’ health for those aged 65 for the period between early-1990s and mid-2010s (by 0.6 year).[62, 67] Similar findings were obtained for individuals at age 15 in 1994–1999 –with a greater increase in life expectancy than health expectancy by 0.9 year.[38] Contradictory findings were produced by a study using the GHS/GLS, which showed that years with self-rated good health at birth increased by 1.3 years more than total life expectancy in 2000–11.[62] However, the response option changed during the observation period, hence the reliability of the findings is questionable.[62] The inconsistencies in the methodology were accounted for statistically in another study, which focused on England only and included a longer period (2000–2014). [70] The study showed that total life expectancy increased more at birth than years with self-rated good health by 0.9.[70] A greater increase in total life expectancy than health expectancy was also found by Jivraj and colleagues, for the period of 1991–2014 among participants aged 25–64.[41]

#### Measures of cognition

A study estimating health expectancy without cognitive impairment between 1991 and 2011 at age 65 found that health expectancy increased more than total life expectancy among females (by 0.8 years).[67] Whereas for males, the number of years without cognitive-impairment lagged behind increase in life expectancy (by 0.3 years).[67]

## Discussion

### Summary of findings

This is the first systematic review of evidence on the joint progress of health and mortality in the UK. We assessed trends of morbidity, based on large population-based surveys and primary/secondary care databases or other routinely collected data. The trends in prevalence of chronic morbidity clearly support the expansion of morbidity scenario for the entire period of 1970s to mid-2010s across all ages, with some evidence pointing towards expansion in coronary heart diseases occurring already from 1950s. Rising prevalence was observed in all studied conditions except for coronary heart disease that appears to have been stable in the period of 2000s-2010s (still supporting expansion of morbidity scenario) and Alzheimer`s disease and other dementias (1989–2013)–reflecting compression of morbidity in severe cognitive impairment (particularly among females).[67] Likewise, the evidence consistently points towards expansion in limiting longstanding morbidity (including illness or disability) and self-reported general health, both at birth and among older people, for the period of 1980s-2010s. For those measures increase in health expectancy lagged behind the increase in total life expectancy. The evidence based on measures of disability, such as daily activities (e.g. mobility, self-care) was inconsistent. It appears that there was compression of morbidity in severe disability (with stable prevalence trends) between early 1980s and mid-1990s. Overall, expansion of morbidity in disability among older population appears to be the more likely scenario in the period 1990s-2010s. The evidence on disability measures in lacking for younger age is lacking, with one study pointing towards expansion of morbidity.

### Comparison with other evidence

Findings from our review were largely consistent with the GBD study providing support for the expansion of morbidity for: diabetes, lung cancer, stroke, asthma, chronic obstructive pulmonary disease, migraine.[20, 21] Inconsistencies were found for Alzheimer`s and other dementias as GBD found no difference in prevalence rates over time (expansion of morbidity), while studies included in our review suggested a decrease (compression of morbidity).[20, 21] This, however, again may be due to different age distribution of the sample (all vs 50-year-olds or older) and definition of ‘caseness’. As far as coronary hearts disease is concerned, GBD found no difference among females (expansion of morbidity) and decrease among males (compression of morbidity) over time, whereas our review suggested increase in both sexes (expansion of morbidity). Overall, our findings seem to be consistent with the GBD study, which found that health expectancy—based on the overall prevalence of a range of health outcomes multiplied by the disability weights—has increased to a lesser extent than total life expectancy (3.2 vs 4.2 years).[20] This finding is true for all EU15+ countries, although the difference between the increase in life expectancy at birth and health expectancy varied from 0.5 years in Greece to 1.7 years in Luxembourg.[20] Chatterji and colleagues also found expansion of chronic morbidity for the period 1991–2011 in worldwide studies, whereas no discernible patterns across countries or within countries over time emerged in disability.[69]

### Explanations for the findings

There are several potential explanations for our findings. The improved survival (e.g., from stroke and some cancers), due to more effective disease management,[19, 39, 40, 49, 54] appears to lead to a higher prevalence of morbidity and an increase in the number of people living with disorders that previously would have been fatal.[20, 21] Although there is some evidence for declining incidence in coronary heart diseases or stroke, these decreases appear to lag behind improvements in survival (see S1 File). For other conditions, such as diabetes, the incidence has increased in the last three decades–hence further expansion of morbidity is expected (S1 File).

It is also likely that more effective screening, combined with greater health awareness—rather than the actual burden of chronic diseases—have contributed to the rising rates. For instance, there has been some concerns that asthma may be currently overdiagnosed in primary care, after years of underdiagnosis.[75] Also, the quality of recording tends to improve over time, particularly after adopting new computer systems, which may lead to higher estimates.[76] Moreover, rising rates of morbidity may be partially caused by programmes incentivising accurate maintenance of registers of patients with diseases such as asthma or diabetes (e.g. the New General Medical Services Contract[77]). However, studies that limited their analysis to services with highly accurate data—as a sensitivity check—tended to find similar trends–for instance in diabetes.[49] Interpretability of trends in diabetes specifically, is also limited by lowering of plasma glucose threshold for diagnoses of diabetes in 2000, which did result in an initial sharp increase in diagnosis in early 2000s.[54] Nonetheless, the rising trends in diabetes have been consistently observed before and after 2000. Moreover, prevalence of diabetes also increased as indicated by haemoglobin A1c (HbA1c), in addition to self-reported diagnosis.[41, 71]

The trends in chronic health should also be considered in the context of trends in risk factors. These, however, are inconsistent over time with some important risk factors decreasing since 1990s, for instance systolic blood pressure, total cholesterol, smoking or heavy drinking,[25, 41, 71, 73, 78] and others increasing–obesity, hypertension and sedentary lifestyle.[25, 41, 71] It also appears that overall there was a greater decline in risk factors more strongly associated with mortality (e.g. smoking) than morbidity (e.g. obesity), hence providing a partial explanation for expansion of morbidity.[79] It is important to note that individuals with less education and of manual social class experienced relatively worse trends in risk factors over time.[65, 73] This has increased the health gap between those of low and high socioeconomic position.[65, 73]

It is also important to note that people`s knowledge and expectations about health may have improved over time, due to better education,[80] which might have raised propensity to report health problems and to have higher expectations from health services. However, empirical evidence testing this hypothesis is lacking. There are still inconsistencies regarding reports of IADL (e.g. independence in shopping) and, more severe, ADL disability (e.g. independence in bathing). Declines in IADLs, should be expected, partially due to improvements in the environment, for instance, wheelchair access or availability of ready-made meals or microwave ovens.[10] However, similar aids are difficult to implement for ADLs.[10] Hence declines in this type of disability may be less likely to occur, unless secondary and tertiary care is more successful in offsetting disabling effects of rising chronic diseases, for which there is currently no evidence.

### Implications

Our review is the first one conducted systematically, which provides a comprehensive and critical insight into the existing evidence. Overall, the rates of morbidity as well as time spent in morbid states, have increased in the last three decades. This is, partially, due to remarkable improvement in survivorship with most chronic conditions. A similar success story is needed in preventing disabling effect of chronic morbidity, as its prevalence is expected to rise,[81] and in delaying the onset of chronic morbidity. Currently there is no evidence for effective practices in either of these processes–with a potential exception for the most severe morbidities, for example related to effective control of hypertension prior to stroke.[43]

A holistic approach to prevention ought to account for an accumulation of health risks across the lifespan–starting in childhood.[7] Hence, an effective approach needs to address modifiable risk factors, which origin in early life–including alcohol consumption, smoking, sedentary lifestyle and diet. Specific recommendations for tackling these problems have been developed. For instance, the National Institute for Health and Care Excellence emphasised the need to recognise the alcohol consumption problem early and act both at the population level (e.g. by reducing marketing of harmful substances) and the individual level (e.g. cognitive-behavioural skills training).[82] Nonetheless, it is important to recognise that risk factors cluster and accumulate to a greater extent among those of low socioeconomic position.[83] People of manual social class or low education lagged behind more advantaged groups in their improvement in life expectancy, and to even a greater extent, in health expectancy–disproportionally extending their time lived with morbidity.[63, 64] These inequalities were also seen at the national level (Scotland having worse outcomes than England) and regional level (Northern England having greater burden of morbidity than Southern England).[53, 57, 58] Again, regional variability was larger in mortality than morbidity.[84] These socioeconomic differences appear to be greater in the rates of risk factors, rather than in disease management.[40, 57, 84] Overall, this emphasises the need for health policies and interventions targeted at particularly vulnerable groups, in order to close the health gap between rich and poor. In addition, these efforts ought to be more focused on morbidity prevention, where the largest inequalities exist, rather than disease management.

As the prevalence of morbidity is projected to increase in the next decade, it is also necessary to consider how the additional years of life can be managed: to ensure good quality of life, and reduce financial consequences of already existing morbidity.[85] For instance, McCormick and colleagues [86] argued that we should move beyond focus on healthcare and pensions, creating more innovative practices leading to harnessing everyday relationships, enabling older individuals to continue paid or unpaid work, encouraging lifelong learning and building environment that helps to connect older people to services, activities, and other people.[86] Such practices have already been successfully implemented around the world and could be adapted to the UK context (e.g. Healthy Ageing Evidence Review [87]).

### Limitations and future research

Research on disability suffers from methodological inconsistencies leading to mixed findings, which has been repeatedly pointed out in the literature.[11, 79] Also, as it is the case in the USA, data on changes in prevalence of disability and functioning problems are scarce and generally limited to the older population.[19] Disability can occur at any time in life, and research on disability at all ages is requisite.[88] More research is needed, which would address limitations of current disability measures, particularly using objectively measured disability as these are not affected by response bias and are more comparable over time. Moreover, disability measures are often based on a single question, thus the use of instruments with finer gradation of disability severity should be more common.[88, 89] This would allow for the testing of dynamic equilibrium theory and better understanding of the reasons underlying disability trends. Furthermore, future research should combine various health indicators obtained within the same individuals, mainly chronic conditions and disability.[46] Such approach would help to understand if disease management also improves one`s quality of life–in addition to prolonging survivorship. Another limitation of the literature was that very few studies included the institutionalised population,[61] as the changes in the prevalence of institutionalisation over time may lead to under-/overestimation of prevalence of certain diseases. A few studies that took that into account did not, however, find any difference in estimates—due to overall low proportion of the institutionalised population.[61, 65]

### Conclusion

The evidence on trends in prevalence of chronic conditions strongly suggests expansion of morbidity. The increasing prevalence of chronic morbidity may to some extent reflect better diagnostics, but as rising trends occur within short studied periods, it is an unlikely explanation for trends. The trends in disability are less conclusive, however even if the length of life with disability remains the same but the length of life needing treatment for disease increases, lifetime health costs will increase unless the cost of health care are reduced.[19] Thus, there is an urgent need for preventative efforts that would delay the onset of morbidity.

## Supporting information

S1 PRISMA Checklist (DOC)

S1 File Trends in incidence of chronic conditions. (DOC)

S2 File Risk of bias assessment tool. (DOC)

S3 File Summary of data source evaluation. (DOCX)
