# Supplementary material for: Post-war (1946-2017) population health change in the United Kingdom: A systematic review
Source: PLoS One. 2019 Jul 3;14(7):e0218991. doi: 10.1371/journal.pone.0218991 (PMC6608959; doi:10.1371/journal.pone.0218991)
Supplement: S1 File — (DOC) [file pone.0218991.s002.doc]

S1 File. Trends in incidence of chronic conditions.

Coronary heart diseases

There is some evidence that age- specific, adjusted or standardised incidence rates of coronary heart disease, have been decreasing since the mid-1960s,[1-6] particularly due to reduced rates of myocardial infarction,[1-3] thus future declines in prevalence are likely. However, it is unlikely that these reduction in the incidence are large enough to compensate for declining cause-specific mortality.[6]

Stroke

The incidence of stroke has been continuously decreasing from early 1980s, mainly among those aged under 75.[7-12] However, this has coincided with even greater improving survivorship hence leading to increased prevalence.[6]

Colorectal/breast cancer

There was no clear trend in incidence of colorectal cancer is concerned between 1970 and 2007.[13-17] The evidence consistently suggested increase of breast cancer across all ages between 1971 to 2007, with simultaneous decline in mortality, [16, 18] thus leading to higher prevalence rates.[14, 16-21]

Asthma

The incidence was found to decrease between 2001 and 2005 (2001: 6.9, CI95% 6.8–7.0; 2005: 5.2, CI95% 5.1–5.3 per 1000 patient-years, p<0.001).[22]

Alzheimer’s and other dementias

Due to decreasing incidence rates, declines in dementia prevalence are projected to continue.[23] Nonetheless, there is some evidence from a large based on data from a public register in Wales that the incidence has been rising among patients who are 75 years and older.[24]

Osteoarthritis

One study, based on Consultations in Primary Care Archive including 11 general practices in North Staffordshire reported increase in incidence of osteoarthritis between 2003 and 2010 from 0.3 to 2.0/1000 persons among those aged 35-44.[25]

Lung cancer

The incidence rates of lung cancer have been stable over this period with increases seen among women and decreases among men, whilst mortality rates have been declining.[2, 26] Incidence peaked among men in late 1970s and has been declining ever since, whereas among women a steady increase has been found.[14-16, 19, 20, 27-30]

Diabetes

The increase in prevalence of diabetes is driven by rising incidence[31-36] and decrease in mortality.[33]

**References**
