# Supplementary material for: Post-war (1946-2017) population health change in the United Kingdom: A systematic review
Source: PLoS One. 2019 Jul 3;14(7):e0218991. doi: 10.1371/journal.pone.0218991 (PMC6608959; doi:10.1371/journal.pone.0218991)
Supplement: S2 File — (DOC) [file pone.0218991.s003.doc]

S2 File. Risk of bias assessment tool.

**MODIFIED NEWCASTLE - OTTAWA QUALITY ASSESSMENT SCALE**

**COHORT STUDIES**

Note: In the comment section briefly justify your answer and explain if anything was done to assess the impact of a potential bias (e.g. by using sensitivity analyses) and mitigate its consequences (e.g. using sampling weights to increase representativeness).

Different sources of bias may apply to different study designs, for instance attrition will apply to population-based surveys, whereas ascertainment bias will be relevant for the studies based on routinely collected data.

**Selection**

1) Representativeness of the sample for the UK population (to consider: selection bias, attrition, inclusion of institutionalised population)

a) Representative of the population

b) Somewhat representative of the population

c) No description (there is not enough information to make the judgement)

Note. Representativeness of the study population is considered in the context of the population of the UK, rather than the community which the sample was drawn from as in the original tool. Nonetheless, if the study includes participants of a certain sex or age, the representativeness is assessed in the context of that sex or age. A study still can be considered as representative if institutionalised population is not included, however this should be noted in the comment section.

Justify your answer:

*2) Demonstration that outcome of interest was not present at start of study (not applicable in studies estimating lifetime prevalence)

a) Yes

b) No

Justify your answer:

*This item only applies to studies on incidence.

**Outcome**

1) Assessment of outcome

a) Independent or blind assessment stated in the paper, or confirmation of the

outcome by reference to secure records (x-rays, medical records, etc.)

b) Record linkage (e.g. identified through ICD codes on database records)

c) Self-report (i.e. no reference to original medical records or x-rays to confirm the

outcome)

d) No description

Provide a brief description, consider any potential sources of bias:

**Comparability of trends**

1) Studies used the same methodology to assess outcome (consider method of collecting data and definition of outcome; answer ‘no’ if either of these were different and provide explanation)

a) Yes

b) No

c) No description

Justify your answer:

Note. Select ‘Yes’ if diagnostic criteria for a given condition changed at the national level over the study period, however it is unclear if this had a direct impact on the data of interest and make a note of it at the second criterion within comparability of trends.

2) Any other potential biases reducing comparability of the trends (consider: ascertainment bias, e.g. introduction of screening programmes or more effective methods of assessment; changing screening criteria; changing demographics of the population; for instance due to migration, which was not accounted for)?

a) Low risk

b) Moderate risk

c) High risk

d) No description

Justify your answer:

Note. Consider if the potential impact of the bias was tested (e.g. sensitivity analysis) or mitigated. Select ‘No description’ if the author does not provide any information on any other potential biases (not related to previous criteria) and the study design is not described in enough detail to make the judgement.
