# Supplementary material for: Post-war (1946-2017) population health change in the United Kingdom: A systematic review
Source: PLoS One. 2019 Jul 3;14(7):e0218991. doi: 10.1371/journal.pone.0218991 (PMC6608959; doi:10.1371/journal.pone.0218991)
Supplement: S3 File — (DOCX) [file pone.0218991.s004.docx]

S3 File. Summary of data source evaluation (see Table 1 & 2 for details).

As summarised in Table 1 and 2, most commonly used primary/secondary care databases (the Health Improvement Network, n=4; the General Practice Research Database, n=4; QRESEARCH, n=2) were large, continuously updated and representative for the United Kingdom.[1-3] These data sources also appeared to have low risk of bias due to outcome assessment as they all passed through regular rigorous validity and reliability checks, thus risk of bias due to outcome assessment was low.[1-3]

Other, considerably smaller or less-established, primary care databases tended to have limited representativeness of the UK population. This includes the Continuous Morbidity Recording project run by Primary Care Clinical Informatics-Research Unit (PCCIU-R)[4] and Diabetes Audit and Research in Tayside[5] (based in Scotland and not representative for the UK population), Doctors' Independent Network (DIN)[6] (estimates in the included study based only on a subset of family practices), Hospital diabetes register in the Poole area (UK)[7] (unlikely to be representative for the general population). Also, there was no detailed information about how the outcome was assessed in the Quality and Outcomes Framework,[8] Poole hospital diabetes register[7] and the Cardiff/the Vale of Glamorgan routine hospital and mortality data.[9]

It is unclear to what extent the methodology used to record and identify cases in primary/secondary care databases and other routinely collected data was consistent over time, as detailed information was rarely available. Nonetheless, there were certain issues that might have had impact on the estimates of trends. For instance, the Health Improvement Network (THIN) database started recording patients information prospectively from 2002,[3] however a few studies based their estimates on information from earlier periods reaching 1996[10-12] when the records were retrieved from other systems. Those might have differed in their quality and use of clinical codes. Davies and colleagues,[10] however found little change in the use of different coronary heart disease Read codes over the period 1996–2002 in their sensitivity analysis. Other studies did not conduct such analysis,[11, 12] but performed a detailed search of records or only used data meeting the quality assurance standards. There were also changes in the coding system within GPRD, from ICD-8 to READ in 1998[13] and the impact of this change on trends is unclear.

The main UK representative population-based surveys used for estimating trends in prevalence and health expectancy were the Health Surveys for England and Scotland (HSE/S) (n=8), the General Household Survey/General Lifestyle Survey (GHS/GLS) (n=10; see Table 4). Other surveys, tended to be limited to older population (Medical Research Council Cognitive Function and Ageing Study; British Regional Heart Study; the English Longitudinal Study of Ageing), or were only representative for small regions rather than general population (general practices in Gloucestershire; general practices in Leicestershire; Arthritis Research Campaign). Nonetheless, representativeness of the population in those studies was counterbalanced by stability in the population that increased confidence in comparability of trends over time.[14-16] Importantly, only three databases included institutionalised population (MRC CFAS study, UK census and study based on information from general practices in Gloucestershire).

As summarised in Table 2, the outcome in most surveys was self-reported, through self-administered questionnaire, which are prone to a recall or desirability bias.[17] In one study, physician-reported survey was used (National Morbidity Survey) [18] and in two studies self-reports were validated with medical records to address these biases (BRHS).[19, 20] Nonetheless, the data collection procedures in key surveys (GHS/GLS; HSE/S; MRC CFA) tended to be standardised, with extensive training for interviewers and reliability checks, thus they were considered as having low risk of bias due to outcome assessment.

The methodology of the studies was rarely identical over time, however in most cases the changes did not appear to introduce any serious biases. For instance, there were changes to the sampling strategy in HSE/S; GHS/GLS; MRC CFAS; Arthritis Research Campaign, which however did not compromise the representativeness of the sample.

| Table 1. The assessment of the risk of bias in primary/secondary care databases and other routinely collected data. | | | | | |
| --- | --- | --- | --- | --- | --- |
| **Database (n studies)** | **Country** | **Representativeness of the UK population** | **Assessment of outcome** | **Risk of bias due to assessment of outcome** | **Any changes to the methodology over time** |
| THIN (n=4)^[10-12, 21]^ | UK | Representative (primary care) | Record linkage | Low | Consistent from 2002; while prospective data collection for THIN started in 2002, the database includes practice data from the date a practice became computerized, which for some of the THIN practices dates back as far as 1987 |
| GPRD (n=4)^[13, 22-24]^ | UK | Representative (primary and secondary care) | Record linkage | Low | Change of coding system from ICD-8 to READ |
| QRESEARCH (n=2)^[25, 26]^ | England | Representative (primary care) | Record linkage | Low | No information |
| QOF (n=1)^[8]^ | UK | Representative (primary care) | Record linkage | No information | No information |
| CMR (n=1)^[4]^ | Scotland | Representative (primary care; unrepresentative of people from very deprived areas) | Record linkage | Low | No information |
| DIN (n=1)^[6]^ | England/ Wales | Representative of age-sex population structure (primary care; northern population slightly under-represented) | Record linkage | Low | No information |
| DARTS (n=1)^[5]^ | Scotland | Representative of the community population (Tayside, Scotland), but unclear to what extent representative for the UK (primary care) | Record linkage | Low | No information |
| Hospital diabetes register (n=1)^[7]^ | England | Representative of the community population (Poole, England), but unclear to what extent representative for the UK | Record linkage | No information | No information |

| Table 1. Continued. | | | | | |
| --- | --- | --- | --- | --- | --- |
| Routine hospital and mortality data (n=1)^[9]^ | Wales | Somewhat representative (mainly urban area) | Record linkage | No information | Differences in case identification (in 1996, patients were identified from a data derived from a primary care audit and a data set created using record linkage on mainly hospital-based sources; in 2004, the audit data were not available, but HbA1c data from general practice and data from a podiatry clinic were available) |
| *Note.* THIN = Health Improvement Network; ICD-8 = International Statistical Classification of Diseases and Related Health Problems 8th Revision; GPRD = General Practice Research Database; QOF = Quality and Outcomes Framework; CMR = Continuous morbidity recording project; DIN = Doctors' Independent Network; DARTS = Diabetes Audit and Research in Tayside. | | | | | |

| Table 2. The assessment of the risk of bias in population-based surveys. | | | | | | |
| --- | --- | --- | --- | --- | --- | --- |
| **Database**  **(n studies)** | **Country** | **Design** | **Representativeness**  **of UK population** | **Assessment of outcome** | **Risk of bias due to assessment of outcome** | **Any changes to the methodology over time** |
| GHS/GLS (n=10)^[8, 27-36]^ | UK | Repeated cross-sectional (GHS)/ prospective longitudinal (GLS) | Representative (no institutionalised population) | Self-reported (CAPI; face-to-face interview) | Low (highly standardised procedures; extensive training for interviewers) | Change of the design from in 2005, from a cross-sectional to a longitudinal format |
| Health Surveys for England and Scotland (n=8)^[8, 18, 27, 37-41]^ | England/ Scotland | Repeated cross-sectional | Representative (no institutionalised population) | Self-reported (CAPI; face-to-face interview) | Low (highly standardised procedures; extensive training for interviewers; reliability checks) | Highly consistent methodology; nonresponse weighting has been incorporated into the weighting strategy |
| MRC CFAS I & II (n=3)^[42-44]^ | England/ Wales | Repeated cross-sectional | Somewhat representative of age 65+ (rural area in East England, mainly white, healthier; includes institutionalised population) | Self-reported (face-to-face interview) | Low (highly standardised procedures; extensive training for interviewers; reliability checks) | Highly consistent methodology; stable diagnostic criteria (algorithmic approach to diagnosis; CFAS I was a two-stage study whereas CFAS II was one-stage, not accounting for the uncertainty introduced in multistage processes leads to overoptimistic confidence intervals) |

| Table 2. Continued. | | | | | | |
| --- | --- | --- | --- | --- | --- | --- |
| **Database**  **(n studies)** | **Country** | **Design** | **Representativeness of UK population** | **Assessment of outcome** | **Risk of bias due to assessment of outcome** | **Any changes to the methodology over time** |
| BRHS (n=2)^[19, 20]^ | UK | Prospective longitudinal | Somewhat representative of middle age men (socioeconomically and geographically representative; mainly white; lack of inner city populations and towns with high mobility) | Self-reported (self-completed questionnaire) and medical records | Low (high agreement between self-report and medical records, but medical records not available for entire period of study) | Somewhat consistent methodology; the wording of questions and coding schemes were the same for each questionnaire; higher attrition among less healthy participants, simple sensitivity analyses suggested that these differences were unlikely to have a significant impact on the estimated prevalence trends; questionnaires were self-completed, with the exception of the questionnaires in 1979 (administered) and 1999 (assistance offered) |
| UK Population census (n=2)^[45, 46]^ | UK | Repeated cross-sectional | Representative (includes institutionalised population) | Self-reported (self-completed questionnaire) | No information | Disability questions varied somewhat between 1991 and 2001 (a Brass relational model used to account for that) |
| General practice in Leicestershire (n=2)^[16, 47]^ | England | Repeated cross-sectional | Somewhat representative of aged 75+ (representative for the Leicestershire population; similar age, sex and social class distribution to England and Wales; includes institutionalised population) | Self-reported (self-completed questionnaire) | Low (standardised procedures; training for interviewers) | Consistent methodology; stable population |
| National Morbidity Survey (n=1)^[18]^ | England/ Wales | Repeated cross-sectional | Representative sample of GPs | Reported by GPs (self-completed form) | No information | No information |

| Table 2. Continued. | | | | | | |
| --- | --- | --- | --- | --- | --- | --- |
| **Database**  **(n studies)** | **Country** | **Design** | **Representativeness of UK population** | **Assessment of outcome** | **Risk of bias due to assessment of outcome** | **Any changes to the methodology over time** |
| ELSA (n=2)^[48, 49]^ | England/ Wales | Prospective longitudinal | Representative (survey weights used; sample refreshments; no institutionalised population) | Cognitive assessment (face-to-face interview) | Low (highly standardised procedures; extensive training for interviewers; reliability checks) | Consistent operational case definition based on standardised assessments of cognition and function was applied (more consistent than clinical assessments amenable to change in diagnostic criteria) |
| BHPS (n=1)^[50]^ | UK | Prospective longitudinal | Representative (no institutionalised population) | Self-reported (face-to-face interviews) | No information | No information |
| Family Resource Survey (n=1)^[51]^ | UK | Repeated cross-sectional | Representative (no institutionalised population) | Self-reported (face-to-face interviews) | Low (highly standardised procedures; extensive training for interviewers) | No information |
| Arthritis Research Campaign (n=1)^[15]^ | England | Repeated cross-sectional | Somewhat representative (limited to the northwest region) | Self-reported (face-to-face interviews and self-administered questionnaire) | No information | Different modes of data collection (study 1: face to face interview vs study 2: self-administered questionnaire); different definitions for identifying pain syndromes over time, however the bias judged to be minimal by authors |
| Randomly selected from lists of GPs (n=1)^[52]^ | UK | Repeated cross-sectional | No information | Self-reported (self-administered questionnaire) | No information | Consistent sampling methods and response rates |

| Table 2. Continued. | | | | | | |
| --- | --- | --- | --- | --- | --- | --- |
| **Database**  **(n studies)** | **Country** | **Design** | **Representativeness of UK population** | **Assessment of outcome** | **Risk of bias due to assessment of outcome** | **Any changes to the methodology over time** |
| General practices in Gloucestershire (n=1)^[14]^ | England | Repeated cross-sectional | Somewhat representative of aged 75+ (representative age and sex structure; only Gloucestershire, England; mainly rural towns; mainly white population; more affluent; includes institutionalised population) | Self-reported (self-administered questionnaire) | No information | Consistent methodology; stable population; institutionalised population only representative in the second survey |
| Annual Population Survey (n=1)^[31]^ | UK | Repeated cross-sectional | Representative (no institutionalised population) | Self-reported (face-to-face interviews) | No information | Used in comparison with GHS/GLS, no information on consistency of the methodology |
| *Note.* GHS/GLS = General Household Survey/General Lifestyle Survey; CAPI = Computer-assisted personal interviewing; MRC CFAS = Medical Research Council Cognitive Function and Ageing Studies; BRHS = British Regional Heart Study; UK = United Kingdom; GP = General Practitioner; ELSA = English Longitudinal Study of Ageing; BHPS = British Household Panel Survey. | | | | | | |

**References**

1. Khan NF, Harrison SE, Rose PW. Validity of diagnostic coding within the General Practice Research Database: a systematic review. British Journal of General Practice. 2010;60(572):e128–e36. doi: 10.3399/bjgp10X483562.

2. Herrett E, Thomas SL, Schoonen WM, Smeeth L, Hall AJ. Validation and validity of diagnoses in the General Practice Research Database: a systematic review. Br J Clin Pharmacol. 2010;69(1):4-14. doi: 10.1111/j.1365-2125.2009.03537.x. PubMed PMID: 20078607; PubMed Central PMCID: PMCPMC2805870.

3. Lewis JD, Schinnar R, Bilker WB, Wang X, Strom BL. Validation studies of the health improvement network (THIN) database for pharmacoepidemiology research. Pharmacoepidemiol Drug Saf. 2007;16(4):393-401. doi: 10.1002/pds.1335. PubMed PMID: 17066486.

4. Simpson CR, Hannaford PC, Williams D. Evidence for inequalities in the management of coronary heart disease in Scotland. Heart. 2005;91(5):630-4. doi: 10.1136/hrt.2004.036723. PubMed PMID: 15831649; PubMed Central PMCID: PMCPMC1768874.

5. Evans JM, Barnett KN, Ogston SA, Morris AD. Increasing prevalence of type 2 diabetes in a Scottish population: effect of increasing incidence or decreasing mortality? Diabetologia. 2007;50(4):729-32. doi: 10.1007/s00125-006-0585-9. PubMed PMID: 17225122.

6. Lusignan S, Sismanidis C, Carey IM, DeWilde S, Richards N, Cook DG. Trends in the prevalence and management of diagnosed type 2 diabetes 1994-2001 in England and Wales. BMC Family Practice. 2005;6(1):13. doi: 10.1186/1471-2296-6-13. PubMed PMID: 15784133; PubMed Central PMCID: PMCPMC1079812.

7. Gatling W, Budd S, Walters D, Mullee MA, Goddard JR, Hill RD. Evidence of an increasing prevalence of diagnosed diabetes mellitus in the Poole area from 1983 to 1996. Diabetic Medicine. 1998;15(12):1015-21. doi: 10.1002/(SICI)1096-9136(1998120)15:12<1015::AID-DIA719>3.0.CO;2-K. PubMed PMID: 9868974.

8. Bhatnagar P, Wickramasinghe K, Wilkins E, Townsend N. Trends in the epidemiology of cardiovascular disease in the UK. Heart. 2016;102(24):1945-52. doi: 10.1136/heartjnl-2016-309573. PubMed PMID: 27550425; PubMed Central PMCID: PMCPMC5256396.

9. Morgan CL, Peters JR, Currie CJ. The changing prevalence of diagnosed diabetes and its associated vascular complications in a large region of the UK. Diabetic Medicine. 2010;27(6):673-8. Epub 2010/06/16. doi: 10.1111/j.1464-5491.2010.02912.x. PubMed PMID: 20546286.

10. Davies AR, Smeeth L, Grundy EM. Contribution of changes in incidence and mortality to trends in the prevalence of coronary heart disease in the UK: 1996 2005. European Heart Journal. 2007;28(17):2142-7. doi: 10.1093/eurheartj/ehm272. PubMed PMID: 17636307.

11. Sharma M, Nazareth I, Petersen I. Trends in incidence, prevalence and prescribing in type 2 diabetes mellitus between 2000 and 2013 in primary care: a retrospective cohort study.[Erratum appears in BMJ Open. 2016;6(5):e010210corr1; PMID: 27147381]. BMJ Open. 2016;6(1):e010210. PubMed PMID: 26769791.

12. Gonzalez EL, Johansson S, Wallander MA, Rodriguez LA. Trends in the prevalence and incidence of diabetes in the UK: 1996-2005. Journal of Epidemiology and Community Health. 2009;63(4):332-6. doi: 10.1136/jech.2008.080382. PubMed PMID: 19240084.

13. Soriano JB, Kiri VA, Maier WC, Strachan D. Increasing prevalence of asthma in UK primary care during the 1990s. International Journal of Tuberculosis and Lung Disease. 2003;7(5):415-21. PubMed PMID: 12757040.

14. Donald IP, Foy C, Jagger C. Trends in disability prevalence over 10 years in older people living in Gloucestershire. Age and Ageing. 2010;39(3):337-42. PubMed PMID: 358767073.

15. Harkness EF, Macfarlane GJ, Silman AJ, McBeth J. Is musculoskeletal pain more common now than 40 years ago?: Two population-based cross-sectional studies. Rheumatology. 2005;44(7):890-95. PubMed PMID: 41511184.

16. Jagger C, Clarke M, Clarke SJ. Getting older--feeling younger: the changing health profile of the elderly. Internation Journal of Epidemiology. 1991;20(1):234-8. Epub 1991/03/01. PubMed PMID: 2066227.

17. Althubaiti A. Information bias in health research: definition, pitfalls, and adjustment methods. J Multidiscip Healthc. 2016;9:211-7. doi: 10.2147/JMDH.S104807. PubMed PMID: 27217764; PubMed Central PMCID: PMCPMC4862344.

18. Scarborough P, Wickramasinghe K, Bhatnagar P, Rayner M. Trends in coronary heart disease 1961-2011. London: British Heart Foundation, 2011.

19. Lampe FC, Morris RW, Whincup PH, Walker M, Ebrahim S, Shaper AG. Is the prevalence of coronary heart disease falling in British men? Heart. 2001;86(5):499-05. PubMed PMID: 32999857.

20. Thomas MC, Hardoon SL, Papacosta AO, Morris RW, Wannamethee SG, Sloggett A, et al. Evidence of an accelerating increase in prevalence of diagnosed Type 2 diabetes in British men, 1978-2005. Diabetic Medicine. 2009;26(8):766-72. doi: 10.1111/j.1464-5491.2009.02768.x. PubMed PMID: 19709145.

21. British Lung Foundation. Lung cancer statistics 2017 [10/05/2017]. Available from: <https://statistics.blf.org.uk/lung-cancer>.

22. Lee S, Shafe AC, Cowie MR. UK stroke incidence, mortality and cardiovascular risk management 1999-2008: time-trend analysis from the General Practice Research Database. BMJ Open. 2011;1(2):e000269. doi: 10.1136/bmjopen-2011-000269. PubMed PMID: 22021893; PubMed Central PMCID: PMCPMC3211058.

23. Soriano JB, Maier WC, Egger P, Visick G, Thakrar B, Sykes J, et al. Recent trends in physician diagnosed COPD in women and men in the UK. Thorax. 2000;55(9):789-94. PubMed PMID: 10950900; PubMed Central PMCID: PMCPMC1745847.

24. Fleming KM, Aithal GP, Solaymani-Dodaran M, Card TR, West J. Incidence and prevalence of cirrhosis in the United Kingdom, 1992-2001: a general population-based study. Journal of Hepatology. 2008;49(5):732-8. doi: 10.1016/j.jhep.2008.05.023. PubMed PMID: 18667256.

25. Simpson CR, Hippisley-Cox J, Sheikh A. Trends in the epidemiology of chronic obstructive pulmonary disease in England: a national study of 51 804 patients. British Journal of General Practice. 2010;60(576):277-84. doi: 10.3399/bjgp10X514729. PubMed PMID: 20594429; PubMed Central PMCID: PMCPMC2894402.

26. Simpson CR, Sheikh A. Trends in the epidemiology of asthma in England: a national study of 333,294 patients. Journal of the Royal Society of Medicine. 2010;103(3):98-106. doi: 10.1258/jrsm.2009.090348. PubMed PMID: 20200181; PubMed Central PMCID: PMCPMC3072257.

27. Lafortune G, Balestat G, the Disability Study Expert Group Members. Trends in Severe Disability Among Elderly People: Assessing the Evidence in 12 OECD Countries and the Future Implications. Paris: Organisation for Economic Co-operation and Development 2007.

28. Jarvis C, Tinker A. Trends in old age morbidity and disability in Britain. Ageing & Society. 1999;19(5):603-27.

29. Jagger C. Trends in life expectancy and healthy life expectancy. Future of an ageing population: evidence review. London: Foresight, Government Office for Science, 2015.

30. Kelly S, Baker A, Gupta S. Healthy life expectancy in Great Britain, 1980–96, and its use as an indicator in United Kingdom Government strategies. Health Statistics Quarterly 07. London: ONS, 2000.

31. Public Health England. Chapter 2: major causes of death and how they have changed. 2017.

32. Jivraj S, Goodman A, Pongiglione B, Ploubidis GB. Living longer but not necessairly healthier: The joint progress of health and mortality in the working age population in England. Manuscript submitted for publication. under review.

33. Bebbington AC, Darton RA. Healthy life expectancy in England and Wales: recent evidence. PSSRU Discussion Paper 1205. Canterbury, Kent: PERSONAL SOCIAL SERVICES RESEARCH UNIT, 1996.

34. Bebbington AC. The Expectation of life without disability in England and Wales. Social Science & Medicine. 1988;27(4):321-6.

35. Smith MP, Olatunde O, White C. Inequalities in disability-free life expectancy by area deprivation: England, 2001–04 and 2005–08. Health Statistics Quarterly. 2010;48:1-22.

36. Robine JM, Ritchie K. Healthy life expectancy: evaluation of global indicator of change in population health. BMJ. 1991;302(6774):457-60. PubMed PMID: 1825931; PubMed Central PMCID: PMCPMC1669345.

37. Hamer M, Kengne AP, Batty GD, Cooke D, Stamatakis E. Temporal trends in diabetes prevalence and key diabetes risk factors in Scotland, 2003-2008. Diabetic Medicine. 2011;28(5):595-8. doi: 10.1111/j.1464-5491.2011.03254.x. PubMed PMID: 21480969.

38. Imkampe AK, Gulliford MC. Increasing socio-economic inequality in type 2 diabetes prevalence--repeated cross-sectional surveys in England 1994-2006. European Journal of Public Health. 2011;21(4):484-90. doi: 10.1093/eurpub/ckq106. PubMed PMID: 20685812.

39. Samaranayakaa S, Gulliforda MC. Trends in cardiovascular risk factors among people with diabetes in a population based study, Health Survey for England 1994–2009. Primary Care Diabetes 2013;7:193–8.

40. Bajekal M. Healthy life expectancy by area deprivation: magnitude and trends in England, 1994-1999. Health statistics quarterly. 2005;(25):18-27. PubMed PMID: 40783246.

41. Martin LG, Schoeni RF, Andreski PM, Jagger C. Trends and inequalities in late-life health and functioning in England. Journal of Epidemiology and Community Health. 2012;66(10):874-80. doi: 10.1136/jech-2011-200251. PubMed PMID: 22147749.

42. Jagger C, Matthews R, Matthews F, Robinson T, Robine JM, Brayne C. The burden of diseases on disability-free life expectancy in later life. Journals of Gerontology - Series A Biological Sciences and Medical Sciences. 2007;62(4):408-14. PubMed PMID: 47065709.

43. Matthews FE, Arthur A, Barnes LE, Bond J, Jagger C, Robinson L, et al. A two-decade comparison of prevalence of dementia in individuals aged 65 years and older from three geographical areas of England: results of the Cognitive Function and Ageing Study I and II. Lancet. 2013;382(9902):1405-12. doi: 10.1016/S0140-6736(13)61570-6. PubMed PMID: 23871492; PubMed Central PMCID: PMCPMC3906607.

44. Jagger C, Matthews FE, Wohland P, Fouweather T, Stephan BC, Robinson L, et al. A comparison of health expectancies over two decades in England: results of the Cognitive Function and Ageing Study I and II. Lancet. 2016;387(10020):779-86. doi: 10.1016/S0140-6736(15)00947-2. PubMed PMID: 26680218; PubMed Central PMCID: PMCPMC4761658.

45. Wohland P, Rees P, Gillies C, Alvanides S, Matthews FE, O'Neill V, et al. Drivers of inequality in disability-free expectancy at birth and age 85 across space and time in Great Britain. Journal of epidemiology and community health. 2014;68(9):826-33. PubMed PMID: 609186684.

46. Congdon P. Modelling changes in small area disability free life expectancy: Trends in London wards between 2001 and 2011. Statistics in Medicine. 2014;33(29):5138-50. PubMed PMID: 600268833.

47. Spiers N, Jagger C, Clarke M. Physical function and perceived health: cohort differences and interrelationships in older people. The Journals of Gerontology Series B, Psychological Sciences and Social Sciences. 1996;51(5):226-33. Epub 1996/09/01. PubMed PMID: 8809007.

48. Ahmadi-Abhari S, Guzman-Castillo M, Bandosz P, Shipley MJ, Muniz-Terrera G, Singh-Manoux A, et al. Temporal trend in dementia incidence since 2002 and projections for prevalence in England and Wales to 2040: modelling study. BMJ. 2017;358:j2856. doi: <http://dx.doi.org/10.1136/bmj.j2856>.

49. Chatterji S, Byles J, Cutler D, Seeman T, Verdes E. Health, functioning, and disability in older adults-present status and future implications. Lancet. 2015;385(9967):563-75. doi: 10.1016/S0140-6736(14)61462-8. PubMed PMID: WOS:000349213600035.

50. Groot W, van den Brink HM. Health-adjusted life expectancy of the British population. Applied Economics. 2008;40(11):1373-86. doi: 10.1080/00036840600820671.

51. Morciano M, Hancock RM, Pudney SE. Birth-cohort trends in older-age functional disability and their relationship with socio-economic status:Evidence from a pooling of repeated cross-sectional population-based studies for the UK. Social Science & Medicine 2015;136-137:1-9. doi: 10.1016/j.socscimed.2015.04.035.

52. Palmer KT, Walsh K, Bendall H, Cooper C, Coggon D. Back pain in Britain: comparison of two prevalence surveys at an interval of 10 years. BMJ. 2000;320:1577–8.
